# Platinum(iv) azido complexes undergo copper-free click reactions with alkynes†
†Electronic supplementary information (ESI) available: Synthesis and characterisation of Pt(i)I complexes, UV-vis, IR spectra, ESI-MS/MS. See DOI: 10.1039/c7dt04183g


**DOI:** 10.1039/c7dt04183g

**Published:** 2018-02-26

**Authors:** Nicola J. Farrer, Gitanjali Sharma, Rachel Sayers, Evyenia Shaili, Peter J. Sadler

**Affiliations:** a Chemistry Research Laboratory , University of Oxford , 12 Mansfield Road , Oxford , OX1 3TA , UK . Email: Nicola.Farrer@chem.ox.ac.uk ; Tel: +44 (0)1865 285155; b Department of Chemistry , University of Warwick , Gibbet Hill Road , Coventry , CV4 7AL , UK

## Abstract

We report our investigations into the first examples of copper-free 1,3-dipolar cycloaddition (click) reactions of electrophiles with a Pt^IV^ azido complex. The Pt-^IV^ azido complex *trans*, *trans*, *trans*-[Pt^IV^(py)_2_(N_3_)_2_(OH)_2_] (**1**) was reactive towards dimethyl acetylenedicarboxylate (DMAD) (**2**), diethyl acetylenedicarboxylate DEACD (**3**), *N*-[(1*R*,8*S*,9*s*)-bicyclo[6.1.0]non-4-yn-9-ylmethyloxycarbonyl]-1,8-diamino-3,6-dioxaoctane (BCN) (**11**) and dibenzocyclooctyne-amine (DBCO) (**12**) resulting in formation of the corresponding mono (a) and bis-substituted (b) complexes. Complexes of **2** undergo further reactions between the Pt centre and the carbonyl group to form **2a′** and **2b′**. This is not seen for the products of the corresponding Pt^II^ azido complex *trans*-[Pt(py)_2_(N_3_)_2_] with acetylene **2**. Novel complexes **2a′**, **2b′**, **11a** and **11b** have been characterised by multinuclear NMR, IR and UV-vis spectroscopy and ESI-MS. These reactions represent new synthetic routes to novel Pt(iv) complexes.

## Abstract 2

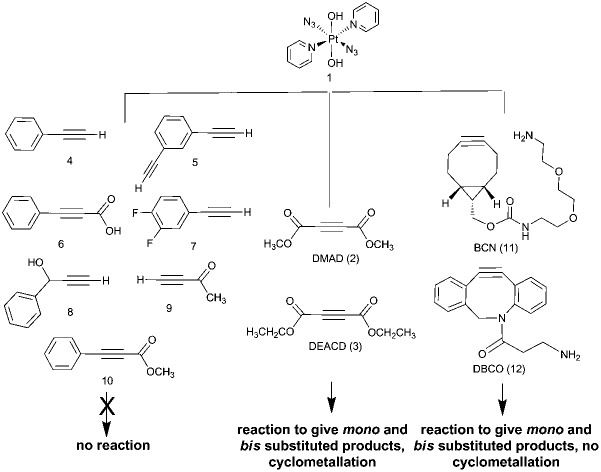
We report our investigations into the first examples of copper-free 1,3-dipolar cycloaddition (click) reactions of electrophiles with a Pt(iv) azido complex.

## Introduction

Copper-free, azide–alkyne cycloaddition (“click”) reactions typically take place under mild conditions^1^,^2^ and provide a tantalising route to modify ligands which are coordinated to a metal centre. Since the conditions are well-tolerated by most functional groups, the reaction can be used to introduce sensitive functionality to complexes at a late stage. By avoiding the use of cytotoxic copper salts it is particularly suitable for complexes with eventual biological applications.^3^ Metal azido complexes have a rich cycloaddition chemistry.^4^–^6^ A range of d-block azido complexes, including those of Mn(i),^7^,^8^ Fe(iii),^9^ Pd(ii),^10^ Pt(ii),^10^–^15^ Rh(iii)^16^ and Au(i),^17^ have been reported to undergo copper-free 1,3-dipolar cycloaddition or “click” reactions with carbon–carbon and carbon-heteroatom functional groups such as alkynes, isocyanides, isonitriles, nitriles, carbon disulphides and isothiocyanates. Electron-deficient alkynes such as dimethyl acetylenedicarboxylate (DMAD) and diethyl acetylenedicarboxylate (DEACD) are relatively reactive: Mo(ii),^16^,^18^ Co(iii),^19^ Fe(iii)^19^,^20^ Ru(ii),^20^–^25^ Pd(ii)^26^–^28^ and Ta^29^ azido complexes all react with DMAD. Strain-promoted azide–alkyne cycloadditions (SPAAC)^30^,^31^ are also an effective method for derivatising azido complexes.^32^ Click reactions between metal azides and alkynes have been used to form Pt(ii) heterometallic arrays with Au(i)^15^ or Re/Rh;^33^ in the development of new catalysts (Ru)^21^ and metalloenzyme inhibitors (Co(iii), Fe(iii), Ni(ii)),^19^ and to synthesise peptide bioconjugates (Au(i)) targeted to mitochondria.^3^ Azolato-bridged platinum complexes show promising anti-cancer activity,^34^ and 1,2,3-triazole ligands themselves have a wide-range of potential applications including in biomedical^35^ and materials chemistry.^36^

**Scheme 1:**
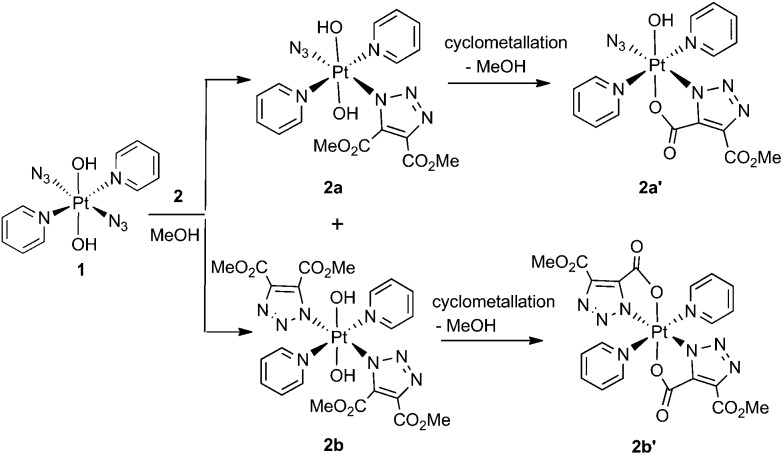
Reaction of **1** with DMAD (**2**).

Our interest in these reactions stems from our investigations of photoactivatable platinum(iv) azido anti-cancer complexes such as *trans*,*trans*,*trans*-[Pt(N_3_)_2_(OH)_2_(py)_2_] (**1**, Scheme 1). Complex **1** is inert in the absence of light, but shows potent cytotoxicity towards cancer cell lines upon irradiation with visible light.^37^ The mechanism of cell death may be due to the formation of a number of different species including azido radicals, nitrenes and singlet oxygen,^38^,^39^ and it is not clear whether both azido groups are necessary for the photocytotoxic effect. The synthetic route to these complexes involves oxidation from Pt^II^ to Pt^IV^ with H_2_O_2_, a step which is incompatible with a number of sensitive functional groups. The capacity for derivatising one (or both) azido groups on a Pt^IV^ centre through click chemistry therefore provides a route to a wide array of functionality, and the reaction itself is biologically compatible. It can be used to produce Pt^IV^ mono azido complexes, a relatively unexplored class of compounds. Whilst there are reports of Pt^II^ azido complexes undergoing click reactions, there are none for Pt^IV^ systems, which are anticipated to react significantly more slowly.

**Fig. 1:**
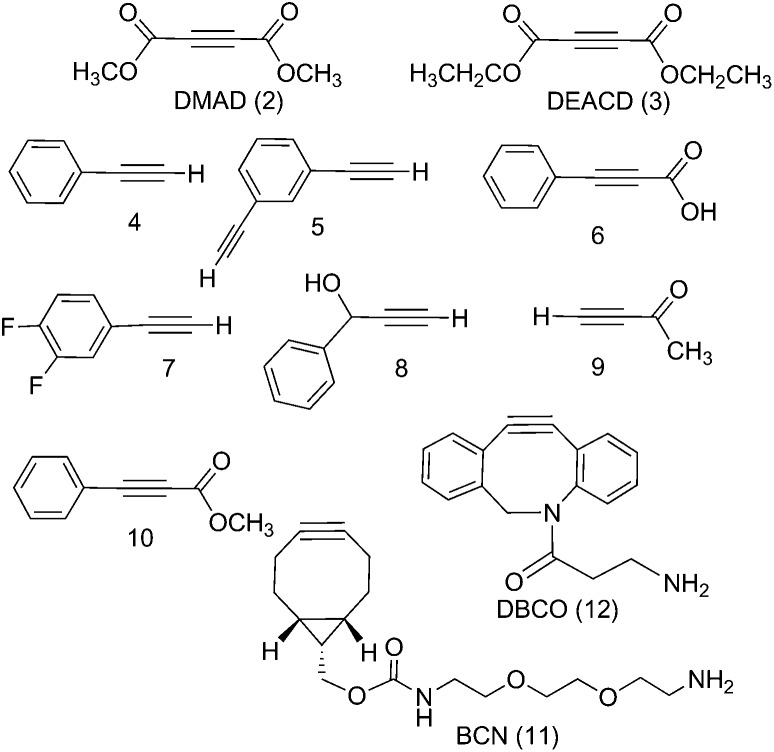
Commercially available alkynes investigated for their reactivity towards *trans*,*trans*,*trans*-[Pt(N_3_)_2_(OH)_2_(py)_2_] (1): DMAD (2), DEACD (3), *N*-[(1*R*,8*S*,9*s*)-bicyclo[6.1.0]non-4-yn-9-ylmethyloxycarbonyl]-1,8-diamino-3,6-dioxaoctane and BCN (11) dibenzocyclooctyne-amine, DBCO (12) were reactive, whereas phenylacetylene (4); 1,3-diethynylbenzene (5); phenylpropiolic acid (6); 3,4-difluorophenylacetylene (7) 1-phenyl-2-propyn-1-ol (8); 3-butyn-2-one (9) and 4-phenyl-3-butyne-2-one (10) were not.

We report the results of our investigation of the reactions of *trans*-[Pt^II^(N_3_)_2_(py)_2_] and *trans*,*trans*,*trans*-[Pt^IV^(N_3_)_2_(OH)_2_(py)_2_] (**1**) with a range of internal and terminal alkynes (Fig. 1) including (to the best of our knowledge) the first cycloaddition reactions of a Pt^IV^ azido complex.

## Results and discussion

We have previously reported a number of *cis*- and *trans*-Pt^IV^ azido complexes of the form [Pt(N_3_)_2_(R)_2_(amine_1_)(amine_2_)_2_] (where R = OH, OAc);^40^–^42^ complex **1** was chosen for cycloaddition investigations since it is readily synthesisable on a large scale, displays good solubility in water and a range of organic solvents and possesses convenient NMR spectroscopic handles. Initial experiments with the Pt^II^ precursor complex *trans*-[Pt(py)_2_(N_3_)_2_] indicated reactivity towards DMAD and DEACD which encouraged us to investigate the reactivity of complex **1**.

### Preparation of the Pt^II^ and Pt^IV^ azido complexes

The Pt^II^ and Pt^IV^ azido complexes were synthesised as previously described.^37^ All reactions and manipulations were carried out so as to avoid unneccessary exposure to light.

### Reactivity of *trans*-[Pt^II^(N_3_)(py)_2_] and **1** towards alkynes

Pt^II^ azide complexes are known to adopt various bonding modes and may also undergo a range of reactions as an alternative to cycloaddition. Ligand substitution – for example, substitution of an azide for an alkyne – instead of the anticipated cycloaddition has been reported for the complex *cis*-[Pt(N_3_)_2_(PPh_3_)_2_] under microwave irradiation with HC≡CR (R = Ph, *p*-MeC_6_H_4_) at 100 °C.^43^ In the reaction of [Pd(PPh_3_)_2_(N_3_)_2_] with DMAD (the Pt^II^ derivative has not been investigated), a triazole-bridged dimer was obtained, which was recrystallised in the presence of PPh_3_ to give the bis substituted species [Pd(PPh_3_)_2_(triazole)_2_].^26^ In light of this product diversity, we considered a number of possible products in our investigations.

Furthermore, for several of the alkynes further reactivity following triazole formation was possible, due to the presence of reactive ligand-based groups. We also considered the possibility of N1–N2 rearrangement of the resulting triazole: several ruthenium azides have reacted with DMAD to produce N2-triazole species,^21^,^24^,^32^,^44^ as confirmed by X-ray crystallography, however, no literature examples of monodentate Pt^II^/Pt^IV^ triazoles could be found for us to draw comparisons with; for those Pt^II^ triazoles with chelating groups on the triazole ligand both N1 and N2 coordination has been previously observed, depending on the influence of the chelating group.^45^

The Pt^II^ complex *trans*-[Pt(N_3_)_2_(py)_2_] reacted with DMAD (**2**) in MeOH (35 °C) to give the mono (*trans*-[Pt(C_6_H_6_N_3_O_4_)(N_3_)(py)_2_]) and bis (*trans*-[Pt(C_6_H_6_N_3_O_4_)_2_(py)_2_]) substituted complexes in which both ester groups remained intact (ESI†). The ^195^Pt NMR resonance of the mono substituted complex *trans*-[Pt(C_6_H_6_N_3_O_4_)(N_3_)(py)_2_] was seen at –2219 ppm (*d*_3_-MeCN), approximately half-way between the starting material *trans*-[Pt(N_3_)_2_(py)_2_] (–2122 ppm, *d*_6_-acetone)^37^ and the bis triazole complex *trans*-[Pt(C_6_H_6_N_3_O_4_)_2_(py)_2_] (–2331 ppm, *d*_6_-acetone). The ^1^H NMR spectrum for the mono-substituted complex showed only one OMe environment, and no nOe correlation was observed between the pyridine protons and the OMe group. The ^14^N NMR spectrum of the bis substituted Pt^II^ complex revealed that the characteristic sharp N_β_ and N_γ_ resonances (where assignment is Pt-N_α_N_β_N_γ_) at 230 ppm and 135 ppm were absent, consistent with loss of azido groups (Fig. S1†). Whilst a characteristic azido absorbance (2043 cm^–1^) was still observed for the mono substituted complex *trans*-[Pt(C_6_H_6_N_3_O_4_)(N_3_)(py)_2_], this IR absorbance was absent in the bis triazole.

We investigated the stability of the Pt^II^ triazole complexes in a number of different solvents; both the mono and bis triazole complexes were unstable in *d*_6_-acetone, CDCl_3_, and *d*_3_-MeCN, slowly converting over time to new species, such that NMR spectroscopic experiments needed to be run shortly after sample preparation.

The Pt^IV^ complex **1** showed no reactivity stirring at 35 °C, up to 7 d, with 5 eq. alkyne towards phenylacetylene (**4**); 1,3-diethynylbenzene (**5**); phenylpropiolic acid (**6**); 3,4-difluorophenylacetylene (**7**) 1-phenyl-2-propyn-1-ol (**8**); 3-butyn-2-one (**9**) and 4-phenyl-3-butyne-2-one (**10**). However, it was reactive in different solvents (including acetone, MeOH, EtOH, MeCN, CHCl_3_ and THF) towards a number of alkynes: dimethyl acetylenedicarboxylate DMAD (**2**), diethyl acetylenedicarboxylate DEACD (**3**), *N*-[(1*R*,8*S*,9*s*)-bicyclo[6.1.0]non-4-yn-9-ylmethyloxycarbonyl]-1,8-diamino-3,6-dioxaoctane, BCN (**11**) and dibenzocyclooctyne-amine DBCO (**12**) (Fig. 1).

### Dimethyl acetylenedicarboxylate (DMAD, **2**) and diethyl acetylenedicarboxylate (DEACD, **3**) products

Complex **1** reacted with **2** in MeOH to produce the cyclometallated species **2a′** (1.1 eq. **2**, 35 °C, 4 d) and **2b′** (5 eq. **2**, 35 °C, 3 d), *via* intermediates **2a** and **2b** (Scheme 1).

The Pt^IV^ complex **1** reacted more slowly than its Pt^II^ precursor *trans*-[Pt^II^(N_3_)_2_(py)_2_] with **2**, taking approximately twice as long under similar conditions to achieve conversion to products, as judged by ^1^H NMR spectroscopy. The initial complex **2a** was detected by HPLC in trace amounts as the [**2a** + Na]^+^ adduct at 636.11 *m*/*z* (model 636.09 *m*/*z*) but was not isolated in sufficient quantity for further analysis. Following isolation, the cyclometallated derivative complex **2a′** was indefinitely stable in D_2_O.

Both ^13^C and ^1^H NMR spectra of **2a′** were consistent with attack of the axial hydroxide on the ester group and elimination of MeOH, with a sharp singlet resonance corresponding to a single methyl group (3H) being observed at 3.78 ppm. The ^13^C NMR spectrum for **2a′** revealed inequivalent triazole ^13^C resonances at 138.4 ppm and 136.1 ppm. The ^195^Pt NMR spectroscopic resonance in D_2_O was 91 ppm lower for **2a′** (873 ppm) than for **1** (964 ppm) indicating increased shielding of the Pt centre.^37^ The ^14^N NMR spectrum of **2a′** (Fig. S2†) was consistent with the proposed structure but afforded little additional structural information. The triazole ^14^N resonances may, like N_α_ in coordinated azide (Pt–N_α_–N_β_–N_γ_) be exceptionally broad, and/or may superimpose with either the N_β_ (229.0 ppm) or the N_γ_/N_py_ (164.6 ppm) resonances, both of which were considerably broader than for **1**.^46^ IR spectroscopy confirmed the presence of azide in the mono substituted products, in which the Pt–N_3_ group gave a sharp IR absorbance *ca.* 2047 cm^–1^. Formation of bis substituted **2b′** resulted in loss of the azido IR peak, compared with the mono substituted **2a′** (Fig. S3†).^47^ Complex **1** also reacted with DEACD (**3**) to produce the corresponding cyclometallated mono (**3a′**) and bis (**3b′**) species, at a slower rate than seen for the reaction with DMAD (**2**).

**Fig. 2:**
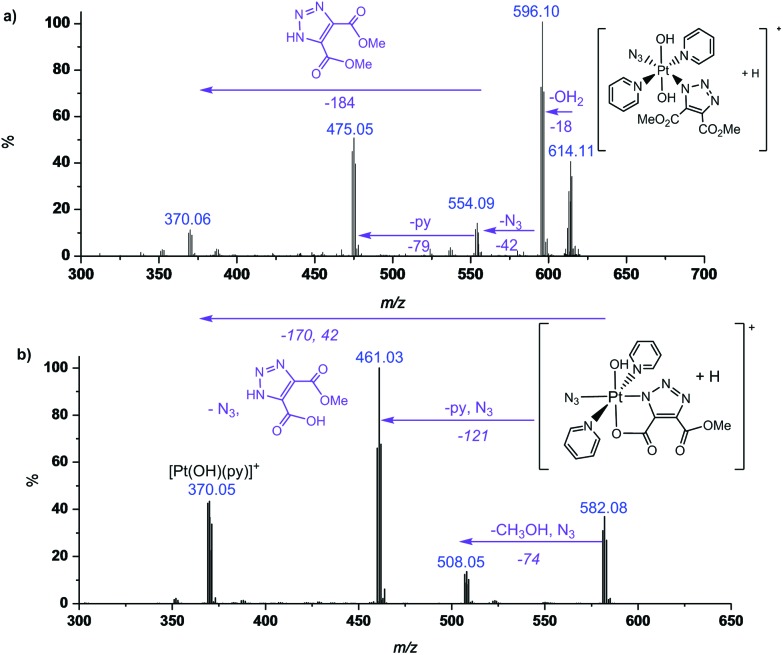
MS/MS of (a) **2a** (614.11 *m*/*z*, [Pt(N_3_)(C_6_H_6_N_3_O_4_)(OH)_2_ (py)_2_ + H]^+^, model 614.10 *m*/*z*) and (b) **2a′** ([Pt(N_3_)(C_5_H_3_N_3_O_4_)(OH) (py)_2_ + H]^+^, model 582.08 *m*/*z*) showing fragmentation through loss of intact ligand for **2a** and cyclometallated ligand for **2a′**.

MS/MS fragmentation studies of DMAD products **2a**, **2a′**, **2b** and **2b′** derivatives revealed that for complexes containing non-cyclometallated triazoles (**2a**, **2b**) these triazole ligands dissociated intact (Fig. 2(a) and S4†). For the cyclometallated complex **2a′** the triazole ligand dissociated by forming the hydroxy species Fig. 2(b); MS/MS of the bis-substituted mono-cyclometallated intermediate between **2b** and **2b′** was also investigated (Fig. S5†); this fragmented through loss of both the cyclometallated and non-cyclometallated triazole ligands.

### Transesterification

**Scheme 2:**
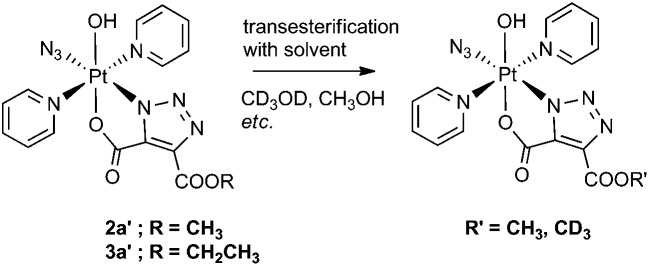
Transesterification of complexes **2a′** and **3a′** with alcoholic solvent.

We observed transesterification between the DEACD triazole ligand and the solvent when the cycloaddition reaction between **1** and **3** was carried out in *d*_4_-MeOH, replacing the remaining –OCH_2_CH_3_ group with –OCD_3_ (Scheme 2). This was detected by ESI-MS as the [M + Na]^+^ adduct [Pt(N_3_)(py)_2_(OH)(N_3_C_3_O_4_D_3_) + Na]^+^ at 607.08 *m*/*z* (model 607.08 *m*/*z*) (Fig. S6†). This species fragmented by MS/MS, showing loss of pyridine and N_3_ to give the fragment [Pt(py)(OH)(N_3_C_3_O_4_D_3_) + Na]^+^ at 486.04 *m*/*z* as well as further fragmentation through loss of the intact cyclised deuterated ligand, giving the [Pt(OH)(py) + Na]^+^ ion at 314.00 *m*/*z*.

A similar transesterification was observed for DMAD derivatives in *d*_4_-MeOH – this could be prevented by conducting reactions and NMR spectroscopic experiments in appropriate solvents.

### Subsequent reactivity

In addition to cyclometallation and transesterification, the complexes also displayed further solvent-dependent reactivity. Although D_2_O and *d*_4_-MeOH solutions of **2a′** were stable (with the exclusion of light) in CDCl_3_ a conversion from **2a′** to **2a′′** was observed. Under extended reaction times (7 d) in MeOH similar reactivity was observed; yellow **2a′** converted completely to off-white **2a′′** (insoluble in D_2_O). The ^195^Pt NMR resonance of **2a′′** in CDCl_3_ (764 ppm) was more shielded than for **2a′** (832 ppm) (ESI†). ^14^N NMR spectra of **2a′** and **2a′′** were not sufficiently diagnostic (Fig. S2†). IR N_3_ azide absorbances (2046 cm^–1^ and 2047 cm^–1^) were essentially the same in strength and frequency for **2a′** and **2a′′** and slightly higher than for the parent complex **1** (2032 cm^–1^).^47^ Compound **2a′′** had the same ^1^H NMR spectral integral ratios between pyridyl and the OCH_3_ group as for **2a′**, indicating that the ester group was retained, but the pyridine H_o_ protons had become deshielded.

Bis-substituted **2b′** was insoluble in D_2_O, MeOH and *d*_6_-acetone but soluble in MeCN and CDCl_3_, giving rise to a ^195^Pt NMR resonance at 840 ppm (CDCl_3_). Complex **2b′** was also unstable in CDCl_3_, converting over a few days to **2b′′**. Complex **2b′′** exhibited inequivalent methyl ^1^H NMR spectroscopic environments with 3H of the singlet corresponding to the remaining OCH_3_ ester groups moving from 3.91 ppm to 3.88 ppm (ESI†). A new pyridyl environment was also observed, with 2H_Pyortho_ protons becoming deshielded (moving from 8.85 ppm to 8.94 ppm) and corresponding new H_m_ and H_p_ resonances overlapping with the existing resonances. Complex **2b′′** was only sparingly soluble in CDCl_3_, precipitating from solution over time. As with **2a′′**, the dominant ESI-MS species were essentially unchanged during the transformation.

Although precedent suggests that a Pt–N1-bound triazole may isomerise to a more thermodynamically stable N2-bound complex^32^ we suggest that the steric requirements of the cyclometallated ring in **2a′** and **2b′** makes N1 to N2 triazole rearrangement unlikely. Further investigations are ongoing to define the precise speciation of **2a′′** and **2b′′**.

### BCN products

**Scheme 3:**
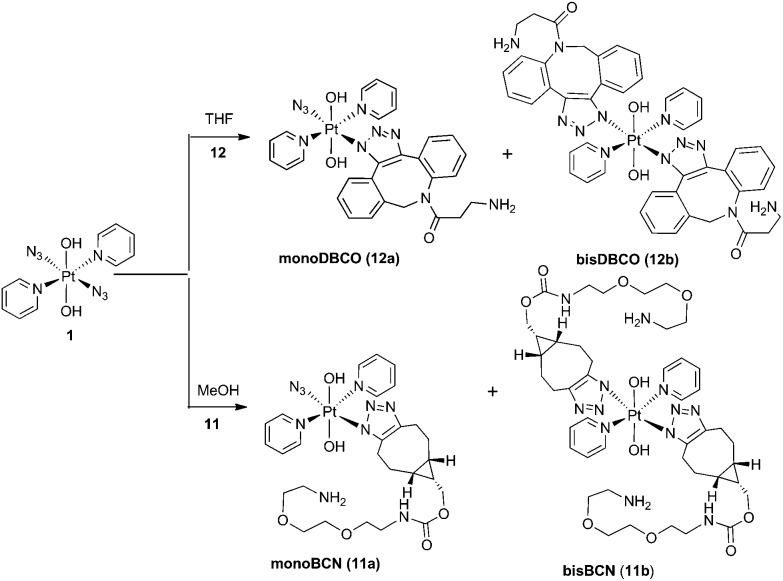
Reaction of **1** with **11** and **12**.

**Fig. 3:**
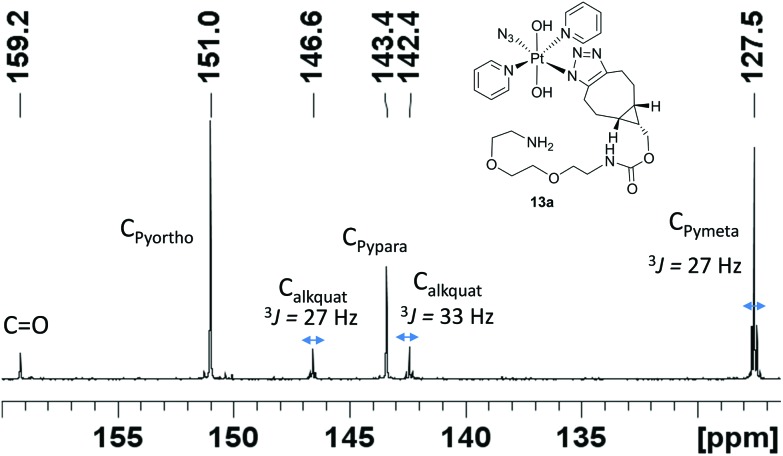
^13^C NMR (d_4_-MeOH) spectrum of **11a** showing ^195^Pt satellites.

Complex **1** reacted with BCN (**11**) in MeOH to give mono (**11a**) and bis (**11b**) substituted products (Scheme 3). Products **11a** and **11b** were soluble in both H_2_O and MeOH. The ^13^C NMR spectra of **11a** revealed the two alkyl carbons at 146.6 ppm and 142.4 ppm with characteristic ^195^Pt satellites and couplings of 27 Hz and 33 Hz, respectively (Fig. 3). This is consistent with the observation that within Pt-pyridyl systems we commonly observe ^3^*J*_PtC_ couplings of this magnitude to C_*meta*_, and we typically do not observe platinum couplings to PyC_*ortho*_ or PyC_*para*_. For **11a**, the inequivalence of these alkyl ^13^C resonances could indicate N1 rather than N2 coordination of the triazole ring (Fig. 3). For **11b** resonances corresponding to the two alkyl carbons were seen at 146.8 ppm and 142.1 ppm, indicating similar coordination as seen for **11a**, but for **11b** the ^195^Pt coupling on these resonances could not be resolved.

MS/MS experiments with [mono-(**11a**) + H]^+^ (796.2 *m*/*z*) showed fragmentation through loss of the various charged (N_3_^–^, OH^–^, triazole *etc.*) and neutral (py) ligands; the triazole ligand did not readily dissociate from the [M + H]^+^ species to give the [M – triazole]^+^ fragment, but a stable fragment [Pt(OH)(py)_2_]^+^ was detected at 370.052 *m*/*z*, indicating it was possible to remove the triazole ligand in association with other ligands. MS/MS experiments with [bis-(**11b**) + H]^+^ (1120.5 *m*/*z*) gave similar fragmentation to the mono species regarding ready loss of H_2_O (1102.473 *m*/*z*) – a common pathway for Pt dihydroxido species (*e.g.***2a** in Fig. 2). Complex [bis-(**11b**) + H]^+^ also showed loss of pyridine ligands to give [Pt(triazole)(triazole – H) + py]^+^ at 1005.423 *m*/*z* and [Pt(triazole)(triazole – H)]^+^ at 926.384 *m*/*z* in which it is assumed one of the amine groups of the BCN ligand provides stabilisation to the Pt centre; for both **11a** and **11b** species the common [Pt(triazole – H)]^+^ fragment was detected around 560.17 *m*/*z*.

### Dibenzocyclooctyne-amine (DBCO) products

Since DBCO **12** was insoluble in MeOH, complex **1** was reacted with **12** in THF, producing both mono- (**12a**) and bis-(**12b**) substituted products (Scheme 3) which showed good agreement with HRMS isotope models (Fig. S8†). Despite concerted efforts with a range of different conditions, the compounds showed poor chromatographic resolution by HPLC and only small quantities of mono-(**12a**) were obtained, sufficient for ESI-MS studies.

MS/MS experiments with [**12a** + H]^+^ (748.2 *m*/*z*) revealed loss of neutral molecules (H_2_O, py) and the residual azido ligand, and fragmentation was consistent with previous MS/MS experiments of complex **1**.^37^ As for the BCN derivatives, the [M – triazole]^+^ species was not detected around 472 *m*/*z*, indicating that the triazole was relatively tightly bound.

### Rates of reaction and purification of products

Compared to Pd^II^, Pt^II^, Ru^II^ 32 and Au azido complexes, the rates of cycloadditions of **1** with alkynes **23** and **11** were relatively slow. Although they did proceed at room temperature, gentle heating (35 °C) was typically employed. To proceed in the absence of a catalyst, these cycloaddition reactions typically require an electron-rich azide and an electron-deficient alkyne. Electron-withdrawing groups on the alkyne lower the level of the LUMO, therefore promoting the reaction. The reactions of **1** with alkynes **2**, **11**, and **12** resulted in a mixture of mono and bis substituted products which in some cases then further converted to additional rearrangement species. The reactions with strained alkynes **11** and **12** proceeded significantly faster (within 24 h at 35 °C) than for the electron deficient alkyne **2** and there was no evidence of decomposition to Pt(0) or cyclometallation. Mono triazole adducts were typically pale yellow, with bis triazole adducts off-white. This was consistent with the UV-vis and IR data, and partial or total loss of the azido group. Attempts to isolate complexes in high purity using standard techniques were aided by mass-directed preparative HPLC. A range of HPLC columns under neutral (and basic – where compatible) conditions were investigated: Atlantis, Hypersil, Sunfire and X-bridge OBD. The best purification results for the complexes were obtained using mass-directed purification on a Waters X-Bridge OBD column, eluting with H_2_O + 0.1% NH_4_OH (pH 9)/MeCN + 0.1% NH_4_OH.

We have previously reported DFT and TDDFT analysis of complex **1**; the absorbance in the UV-vis region is dominated by ^1^LMCT (N_3_ → Pt) and mixed ^1^LMCT/^3^IL (OH → Pt, N_3_; IL = interligand) transitions.^37^ UV-vis spectroscopy of the click products were consistent with partial (mono) and complete loss (bis) of the azido absorbances, compared to the starting complex **1** (Fig. S7†) and resulted in a reduced absorption *ca.* 300 nm.

## Experimental

### General procedures

**Materials.** K_2_[PtCl_4_] was purchased from Precious Metals Online. HPLC-grade solvents and Millipore filtered H_2_O were used for the preparation and purification of compounds by HPLC. All other reagents were purchased from Sigma-Aldrich and used as received. (IM) indicates use of a nylon syringe filter (pore size 0.2 μM). All manipulations were carried out under reduced lighting and solutions were prepared stored and handled with minimal exposure to light. **NMR spectroscopy.** Due to the potential photosensitivity of the compounds, amberised NMR spectroscopy tubes (Goss Scientific) were used. ^**13**^**C NMR:** acquired on a Bruker AVII 500 MHz spectrometer equipped with a *z*-gradient triple resonance inverse ^1^H/^19^F(^13^C) TXI probe and referenced internally to residual solvent where possible or externally to TMS in CDCl_3_. All other NMR spectra were acquired on a Bruker AVIIIHD 500 MHz (^1^H: 500.13 MHz), a Bruker AVIIIHD 400 nanobay, or a Bruker AV111-600 spectrometer at 298 K and processed using Topspin 3.2. *J* values are quoted in Hz. All chemical shift (*δ*) values are given in parts per million. ^**1**^**H NMR:** chemical shifts were referenced to residual solvent. ^**195**^**Pt NMR:** chemical shifts were externally referenced to K_2_PtCl_6_ in 1.5 mM HCl in D_2_O (*δ* 0 ppm) using parameters as previously described: for spectra of Pt^IV^ species directly bonded to quadrupolar ^14^N, typical parameters used were *d*_1_ = 0 s, TD 2k, DE 10 μs, 256k scans.^37^ Data were processed with a LB of 50 Hz. ^**14**^**N NMR:** chemical shifts were externally referenced to [^14^N]NH_4_Cl (1.5 M) in 1 M HCl with a D_2_O coaxial insert and processed with a qfil baseline correction. **Mass spectrometry: low resolution ESI-MS**: obtained with a Waters Micromass LCT Premier XE spectrometer. **HRMS:** obtained with a Thermofisher Exactive Plus with a Waters Acuity UPLC system. **MS/MS experiments**: were performed on an Acuity UPLC in flow injection analysis mode, equipped with a Waters Xevo G25 QTOF. All MS data were processed using MassLynx 4.0. **HPLC:** were performed with a Waters Autopurification system, equipped with a Waters X-Bridge OBD semi-prep column (5 μm, 19 mm × 50 mm), with an injection loop of 1 ml, eluting with H_2_O + 0.1% NH_4_OH (pH 9)/MeCN + 0.1% NH_4_OH. The crude samples (in H_2_O/MeCN) were filtered (nylon, 0.2 μm) and injected in 750 μL aliquots, with mass-directed purification with an ACQUITY QDa performance mass spectrometer. **UV-visible absorption spectra** were acquired with a T60U Spectrometer PG Instruments Ltd using UVWin Software or the Waters HPLC. **Elemental microanalyses** were performed by Stephen Boyer at the London Metropolitan University.

## Materials and methods

### Synthetic procedures

The Pt^II^ complex *trans*-[Pt^II^(N_3_)_2_(py)_2_] and **1** were prepared in two steps from K_2_PtCl_4_, *via trans*-[Pt^II^Cl_2_(py)_2_]. H_2_O_2_ oxidation of *trans*-[Pt^IV^(N_3_)_2_(py)_2_] gave *trans*,*trans*,*trans*-[Pt^IV^(N_3_)_2_(OH)_2_(py)_2_ (**1**) which was purified by HPLC before use. Analytical data was consistent with previous reports.^37^ UV-Vis spectra and data concerning reactivity of *trans*-[Pt^II^(N_3_)_2_(py)_2_] are given in the ESI.†

***Caution!*** No problems were encountered during this work, however heavy metal azides are known to be shock sensitive detonators, therefore it is essential that platinum azides compound are handled with care.

### Pt^IV^ complexes

#### Cycloadditions with DMAD (**2**)

##### Cyclometallated trans,trans,trans-[Pt(N_3_)(C_5_H_3_N_3_O_4_)(OH)(py)_2_] (**2a′**)

DMAD (**2**, 14.3 μl, 0.117 mmol) in MeOH (2 ml) was added to **1** (50 mg, 0.106 mmol) in MeOH (3 ml). The solution was stirred at 35 °C for 4 d before being placed on ice. Product **2a′** was isolated as a yellow precipitate by filtration and rinsed with cold H_2_O, MeOH and diethyl ether (29 mg, 0.05 mmol, 47%).

^**1**^ **H NMR** (400 MHz, D_2_O) *δ*: 8.71 (d, ^3^*J*^1^_H^195^Pt_ = 25, ^3^*J*_HH_ = 6, 4H, H_o_) 8.25 (t, ^3^*J*_HH_ = 7, 2H, H_p_), 7.76 (dd, ^3^*J*_HH_ = 7, ^3^*J*_HH_ = 7, 4H, H_m_), 3.87 (s, 3H, OMe).

^**195**^ **Pt NMR** (107 MHz, D_2_O) *δ*: 873 (PWHH 670 Hz).

^**14**^ **N NMR** (29 MHz, D_2_O) *δ*: 288.6 (N_2_ gas), 229.0 (N_β_), 164.6 (broad, N_γ/py_). N_α_ not seen.

^**13**^ **C NMR** (126 MHz, D_2_O) *δ*: 166.6 (C_ester_), 161.5 (C_ester_), 148.9 (C_pyortho_), 143.3 (C_pypara_), 138.4 (C_triazole_), 136.1 (C_triazole_), 127.8 (^3^*J*_^13^C^195^Pt_ = 25, C_pymeta_), 52.9 (C_alkyl_).

^**1**^ **H NMR** (400 MHz, CDCl_3_) *δ*: 8.98 (d, ^3^*J*^1^_H^195^Pt_ = 25, ^3^*J*_HH_ = 6, 4H, H_o_), 8.14 (t, ^3^*J*_HH_ = 7, 2H, H_p_), 7.72 (dd, ^3^*J*_HH_ = 7, ^3^*J*_HH_ = 7, 4H, H_m_), 1.64 (br) (with peaks corresponding to **2a′′** growing in over time).

^**195**^ **Pt NMR** (107 MHz, CDCl_3_) *δ*: 832 (with peak at 767 ppm corresponding to **2a′′** growing in over time).

**ESI-MS** (MeOH, M = *trans*,*trans*,*trans*-[Pt(N_3_)(C_5_H_3_N_3_O_4_)(OH)(py)_2_]) *m*/*z*: 1185.12 ([2 M + Na]^+^ calcd 1185.14); 1163.14 ([2M + H]^+^ calcd 1163.15); 604.05 ([M + Na]^+^ calcd 604.06); 582.07 ([M + H]^+^ calcd 582.08).

**HRMS** (MeOH) *m*/*z*: 582.0808 ([M + H]^+^, C_15_H_15_N_8_O_5_Pt calcd 582.0766).

**IR** (solid) *ν* cm^–1^: 3465, 3108, 3074, 2046 (*ν*_asym_N_3_), 1732, 1674, 1611, 1538, 1460, 1437, 1389, 1337, 1254, 1211, 1197, 1127, 1078, 1018, 810, 773, 690.

**Elemental microanalysis:** Calc. C_15_H_14_N_8_O_5_Pt (581.07 g mol^–1^): C, 30.99; H, 2.43; N, 19.27. Found: C, 31.15; H, 2.46; N, 19.10.

##### Bis cyclometallated bis trans-[Pt(C_5_H_3_N_3_O_4_)_2_(py)_2_] (**2b′**)

DMAD (65.2 μl, 0.53 mmol) in MeOH (2 ml) was added dropwise to *trans*,*trans*,*trans*-[Pt(N_3_)_2_(OH)_2_(py)_2_] (50 mg, 0.11 mmol) in MeOH (3 ml). The solution was stirred at 35 °C for 3 days, put on ice and the resulting white compound **2b′** isolated by filtration (15.5 mg, 0.02 mmol, 21%).

^**1**^ **H NMR** (400 MHz, CDCl_3_) *δ*: 8.87 (d, ^3^*J*^1^_H^195^Pt_ = 22, ^3^*J*_HH_ = 6 Hz, 4H, H_o_) 8.10 (t, ^3^*J*^1^_H^1^H_ = 6, 2H, H_p_) 7.62 (t, ^3^*J*^1^_H^1^H_ = 6, 4H, H_m_) 3.91 (s, 6H, OMe).

^**195**^ **Pt NMR** (107 MHz, CDCl_3_) *δ*: 840.

^**13**^ **C NMR** (125 MHz, CDCl_3_) *δ*: 162.7 (C_estercyclo_), 160.2 (C_esterOMe_), 153.4, 149.4 (C_o_), 143.4 (C_p_), 139.3 (C_alkene_), 132.7 (C_alkene_), 128.3 (C_m_), 52.6 (C_alkyl_).

**ESI-MS** (MeOH) (M = *trans*-[Pt(C_5_H_3_N_3_O_4_)_2_(py)]) *m*/*z*: 1405.09 ([2 M + Na]^+^ calcd 1405.14); 1383.12 ([2 M + H]^+^ calcd 1383.15); 714.04 ([M + Na]^+^ calcd 714.06); 692.06 ([M + H]^+^ calcd 692.08).

**HRMS** (MeOH) *m*/*z*: 714.06298 ([M + Na]^+^ C_20_H_16_N_8_O_8_PtNa calcd 714.0631). **IR***ν* cm^–1^: 3112, 2051, 1732, 1613, 1541, 1486, 1462, 1436, 1330, 1235, 1169, 1062, 1019, 835, 812, 690.

**Elemental microanalysis** Calc. C_20_H_16_N_8_O_8_Pt (691.07 g mol^–1^): C, 34.73; H, 2.33; N, 16.21. Found: C, 34.65; H, 2.28; N, 16.14.

#### Cycloadditions with BCN (**11**)

BCN (**11**, 25 mg, 0.077 mmol, 1 eq.) was added to **1** (40 mg, 0.085 mmol) in MeOH (5 ml) and the reaction stirred for 16 h at 35 °C. The volume was reduced to 1 ml by rotary evaporation, filtered and purified by HPLC. Products **11a** (796.2 *m*/*z*) and **11b** (1119.5 *m*/*z*) and unreacted **1** were isolated and the solvent removed by freeze-drying.

#### Monosubstituted *trans*,*trans*,*trans*-[Pt(N_3_)(C_17_H_28_N_5_O_4_)(OH)_2_(py)_2_] (**11a**)

Isolated as a pale yellow solid (18 mg, 18%). ^**1**^**H NMR** (500 MHz, *d*_4_-MeOH) *δ*: 8.80 (dd, ^3^*J*^1^_H^195^Pt_ = 27, ^3^*J*_HH_ = 7, 4H, H_Pyortho_), 8.22 (t, ^3^*J*_HH_ = 7, 2H, H_Pypara_), 7.72 (dd, ^3^*J*_HH_ = 7, ^3^*J*_HH_ = 7, 4H, H_Pymeta_), 3.99 (d, 2H, C*H*_2_OC(O)NH), 3.64 (s, 4H), 3.54 (m, 4H), 3.30 (m, 2H, obscured by solvent), 3.00 (t, 1H), 2.90 (m, 1H), 2.82 (m, 2H), 2.68 (m, 1H), 2.31 (m, 1H), 2.17 (m, 1H), 1.42 (m, 1H), 1.32 (m, 1H), 1.13 (m, 1H, C*H*CH_2_OC(O)NH), 1.04 (s, 1H), 0.81 (m, 2H, H_3mring_). ^195^Pt NMR (129 MHz, *d*_4_-MeOH) *δ*: 842. ^13^C NMR (126 MHz, *d*_4_-MeOH) *δ*: 159.2 (*C*=O), 151.0 (C_Pyortho_), 146.6 (*J*^13^_C^195^Pt_ = 27, C_alkquat_), 143.4 (C_Pypara_), 142.4 (*J*^13^_C^195^Pt_ = 33, C_alkquat_), 127.5 (t, ^3^*J*^13^_C^195^Pt_ = 27, C_Pymeta_), 72.9, 71.32, 71.27, 71.0, 63.7 (*C*H_2_OC(O)NH), 41.9, 41.7, 26.5, 25.1, 24.2, 23.5, 22.0, 21.7, 19.3. ESI-MS (MeOH) *m*/*z (*M = *trans*,*trans*,*trans*-[Pt(N_3_)(C_17_H_28_N_5_O_4_)(OH)_2_(py)_2_]): 398.63 [M + 2H]^2+^ calcd C_27_H_42_N_10_O_6_Pt: 398.64439, 796.25 ([M + H]^+^ calcd C_27_H_41_N_10_O_6_Pt: 796.2853), 819.26 ([M + Na]^+^ calcd C_27_H_40_N_10_NaO_6_Pt: 819.2629). HRMS (MeOH) *m*/*z*: 796.284 ([M + H]^+^ calcd C_27_H_41_N_10_O_6_Pt: 796.285).

MS/MS (796.2) (*d*_4_-MeOH) *m*/*z*: 778.276 ([M – OH]^+^ C_27_H_39_N_10_O_5_Pt, calcd 778.273), 718.252 ([M + H – py]^+^ C_22_H_36_N_9_O_6_Pt, calcd 718.242), 657.224 ([M + H – N_3_, py, OH]^+^ C_22_H_34_N_6_O_5_Pt, calcd 657.227), 639.212 ([M – H_2_O_2_, N_3_, py]^+^, C_22_H_32_N_6_O_4_Pt, calcd 639.211), 560.174 ([M – H_2_O_2_, N_3_, 2py, H]^+^, C_17_H_27_N_5_O_4_Pt, calcd 560.172), 370.055 ([Pt(OH)(py)_2_]^+^, C_10_H_11_N_2_OPt calcd 370.055).

IR (MeOH-*d*_4_) cm^–1^: 3361 (br), 2920, 2044 (*ν*_asym_N_3_, strong), 1695, 1613, 1543, 1457, 1264, 1211, 1104, 1077, 1020, 769, 690.

#### Bis BCN (**11b**)

Isolated as an off-white solid (10 mg, 12%). ^1^H NMR (500 MHz, *d*_4_-MeOH) *δ*: 8.52 (m, ^3^*J*^1^_H^195^Pt_ = 27, ^3^*J*_HH_ = 6, 4H, 4H_Pyortho_), 8.20 (t, ^3^*J*_HH_ = 7, 2H, H_Pypara_), 7.65 (dd, ^3^*J*_HH_ = 7, ^3^*J*_HH_ = 7, 4H, H_Pymeta_), 4.01 (m, 4H), 3.68 (m, 2H), 3.64 (m, 8H), 3.54 (m, 6H), 3.45 (m, 2H), 3.29 (m, 2H, obscured by solvent), 3.03 (m, 2H), 2.93 (m, 2H), 2.83 (m, 2H), 2.73 (m, 2H), 2.42 (m, 2H), 2.30 (m, 2H), 2.19 (m, 2H), 1.41, 1.15, 1.07, 0.93, 0.79 (m, 12H). ^195^Pt NMR (129 MHz, *d*_4_-MeOH) *δ*: 782.

^13^C NMR (126 MHz, *d*_4_-MeOH) *δ*: 159.3 (*C*=O), 151.3 (C_Pyortho_), 146.8 (m), 143.7, 142.1 (m), 127.8 (^3^*J*^13^_C^195^Pt_ = 27, C_Pymeta_),71.5(m), 71.3(m), 71.0(m), 69.2, 63.7 (m), 43.9, 41.7(m), 26.5(m), 25.1(m), 24.3(m), 23.7 (m), 22.0(m), 21.5(m), 19.4(m).

ESI-MS (MeOH) *m*/*z* (M = *trans*,*trans*,*trans*-[Pt (C_17_H_28_N_5_O_4_)_2_(OH)_2_(py)_2_]): 560.74 ([M + 2H]^2+^ C_44_H_68_N_12_O_10_PtH_2_ calcd 560.7468), 1120.48 ([M + H]^+^, C_44_H_69_N_12_O_10_Pt, calcd 1120.4902). HRMS (MeOH) *m*/*z*: 1120.4933 [M + H]^+^, C_44_H_69_N_12_O_10_Pt, calcd 1120.4902.

**MS/MS** (1120.5) *d*_4_-MeOH *m*/*z*: 1102.472 ([M – OH]^+^, C_44_H_67_N_12_O_9_Pt, calcd 1102.484), 1067.439, 1005.423 ([M – H_2_O_2_, py, H]^+^ C_39_H_60_N_11_O_8_Pt, calcd 1005.430), 926.383 ([M – H_2_O_2_, 2py, H]^+^, C_34_H_55_N_10_O_8_Pt, calcd 926.384), 560.167 ([M – triazole, H_2_O_2_, 2py, H]^+^, C_17_H_27_N_5_O_4_Pt, calcd 560.172).

IR (MeOH-*d*_4_) cm^–1^: 3366, 2482, 2244, 2072, 1120, 973, 822.

#### Cycloadditions with DBCO (**12**)

Dibenzocyclooctyne-amine (**12**, 20 mg, 0.04 mmol) and **1** (45 mg, 0.04 mmol) were dissolved in THF (15 ml) and stirred at 35 °C for 16 h, giving a yellow solution which was filtered, and the solvent removed under reduced pressure. The solid was reconstituted in 2 ml of 50 : 50 MeCN : H_2_O and purified by HPLC.

### Monosubstituted *trans*,*trans*,*trans*-[Pt(N_3_)(C_18_H_16_N_5_O)(py)_2_(OH)_2_]

DBCO complex **12a** was isolated as a yellow solid by HPLC (4 mg, 7% yield).

**ESI-MS** (MeOH) *m*/*z*: (M = *trans*,*trans*,*trans*-[Pt(N_3_)(C_18_H_16_N_5_O)(py)_2_(OH)_2_]): 748.18 ([M + H]^+^, C_28_H_29_N_10_O_3_Pt calcd 748.21), 770.13 ([M + Na]^+^ C_28_H_28_N_10_O_3_PtNa calcd 770.19 *m*/*z*). **HRMS** (MeOH) *m*/*z*: 748.2067 ([M + H]^+^, C_28_H_29_N_10_O_3_Pt calcd 748.2066).

MS/MS (748.2) *d*_4_-MeOH *m*/*z*: 730.197 ([M – OH]^+^ C_28_H_29_N_10_O_3_Pt, calcd 730.197), 712.186, 688.183 ([M – N_3_, H_2_O]^+^, C_28_H_27_N_7_O_2_Pt calcd 688.187), 669.171 ([M – py + H]^+^ C_23_H_24_N_9_O_3_Pt, calcd 669.164), 609.146 ([M – N_3_, H_2_O, py]^+^, C_23_H_22_N_6_O_2_Pt, calcd 609.148), 590.133 ([M – 2py + H]^+^ C_18_H_19_N_8_O_3_Pt, calcd 590.125). **IR***v* cm^–1^ (*d*_4_-MeOH): 3378(br), 2980, 2493(br), 2047 (*ν*_asym_N_3_), 1637, 1613, 1479, 1457, 1212, 1117, 1076, 1019, 971, 765, 689.

### Bis-substituted *trans*,*trans*,*trans*-[Pt(C_18_H_16_N_5_O)_2_(OH)_2_(py)_2_]

Complex **12b** was isolated as an off-white solid by HPLC (3 mg, 4% yield).

**ESI-MS** (MeOH) *m*/*z*: (M = *trans*,*trans*,*trans*-[Pt(C_18_H_16_N_5_O)_2_(OH)_2_ (py)_2_]): 1024.33 ([M + H]^+^ C_46_H_44_N_12_O_4_PtH, calcd 1024.33), 512.67 ([M + 2H]^2+^ C_46_H_44_N_12_O_4_PtH_2_, calcd 512.67). **HRMS** (MeOH) *m*/*z*: 1024.3335 ([M + H]^+^, C_46_H_44_N_12_O_4_PtH calcd 1024.3285). **IR***v* cm^–1^ (*d*_4_-MeOH): 3346(br), 2479(br), 2216, 2071, 1120, 972, 822.

## Conclusions

**Fig. 4:**
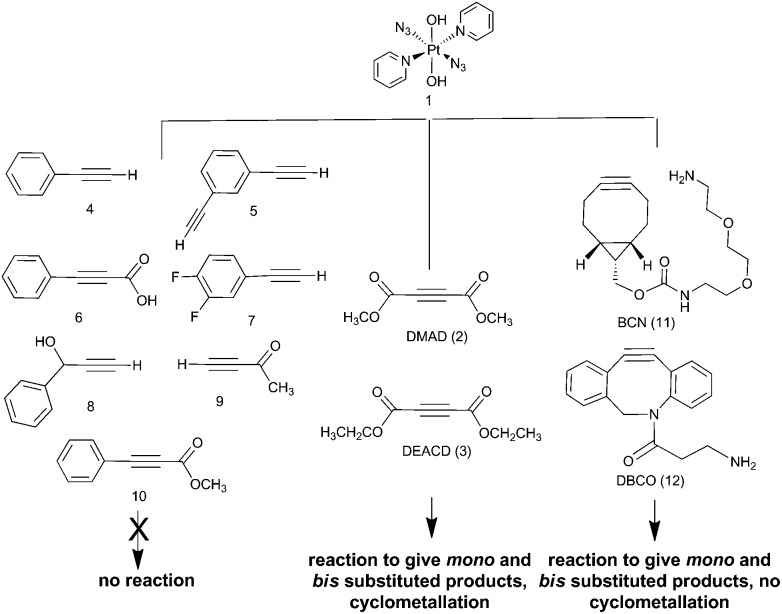
Summary of reactivity observed for complex **1**.

The reactions of azido Pt^IV^ complex **1** with acetylenes **2**, **3**, **11** and **12** represent the first examples of Pt^IV^ azido cycloaddition reactions. The reactions take place under mild conditions in the absence of a catalyst, enabling modification of ligands which are already coordinated to a metal centre, and providing a route to monoazido complexes. Complex **1** did not show any reactivity towards compounds **4–9**; we suggest this is due to the alkynes being insufficiently electron deficient for the reaction to occur (Fig. 4).

For reactions of **1** with **2** and **3** there is evidence that Pt cyclometallates with the new triazole ligand *via* the ester group, displacing MeOH or EtOH respectively. This reactivity is not observed for the Pt^II^ analogue *trans*-[Pt(N_3_)_2_(py)_2_]. Whilst the cyclometallated complex **2a′** was stable in D_2_O, in other solvents there is evidence for subsequent reactivity. In contrast, derivatives of complex **1** with cyclooctynes **11** and **12** did not show any obvious subsequent reactivity following formation of the triazole ligand.

Whilst use of electron withdrawing groups is an effective strategy to promote copper-free cycloadditions, for Pt^IV^ complexes the proximity of these reactive groups to the Pt centre can result in subsequent reactivity. Strain-promoted cycloadditions appear to be a promising alternative strategy, and we are currently investigating appropriate cyclooctynes to further investigate this chemistry.

## Conflicts of interest

There are no conflicts of interest to declare.

## Supplementary Material

Supplementary information
