# Supplementary material for: Platinum(iv) azido complexes undergo copper-free click reactions with alkynes
Source: Dalton Trans. 2018 Feb 26;47(31):10553–60. doi: 10.1039/c7dt04183g (PMC6083821; doi:10.1039/c7dt04183g)
Supplement: Supplementary file 1 [file DT-047-C7DT04183G-s001.pdf]

Electronic Supplementary Material (ESI) for Dalton Transactions.
This journal is © The Royal Society of Chemistry 2018

Pt^{IV} azido complexes undergo copper-free click reactions with alkynes

Electronic Supplementary Information

Nicola J. Farrer,[a*] Gitanjali Sharma,[a] Rachel Sayers,[a] Evyenia Shaili,[b] Peter J. Sadler[b]

Synthesis and Characterisation

***Mono*-substituted *trans*-[Pt($C_6H_6N_3O_4$)(N_3)(py)$_2$]**

Dimethyl acetylenedicarboxylate (**2**, 30.9 µl, 0.25 mmol) in MeOH (5 ml) was added dropwise to *trans*-[Pt(N_3)$_2$(py)$_2$] (100 mg, 0.23 mmol) in MeOH (50 ml). The solution was stirred at 35°C for 2 days and then reduced to dryness. The solution was resuspended in minimal MeCN, filtered (IM) and purified by HPLC under neutral conditions to give the monosubstituted complex as a pale yellow solid (43 mg, 0.07 mmol, 32 %).

^{1}H NMR (400 MHz, d_3-MeCN) δ: 8.73 (d, 4H, $^3J_{HH}$ = 7, $^3J_{HPt}$ = 38, H$_o$), 7.97 (dd, 2H, $^3J_{HH}$ = 7, H$_p$), 7.49 (dd, $^3J_{HH}$ = 7, H$_m$), 3.76 (s, OMe, 6H). ^{195}Pt NMR (86 MHz, d_3-MeCN) δ: -2219 (W ½ 1150 Hz). **ESI-MS** (MeOH) (M = *trans*-[Pt($C_6H_6N_3O_4$)(N_3)(py)$_2$]) *m/z*: 602.111 ([M + Na]$^+$, calcd 602.084); 580.125 ([M + H]$^+$ calcd 580.102) **HRMS** (MeOH) *m/z*: 580.1020 ([M + H]$^+$, $C_{16}H_{17}N_8O_4Pt$, calcd. 580.1015). **IR** ν cm^{-1}: 2043 (N_3), 1728, 1452, 1289, 1172, 1090, 769, 693.

***Bis*-substituted *trans*-[Pt($C_6H_6N_3O_4$)$_2$(py)$_2$]**

Dimethyl acetylenedicarboxylate (**2**, 141 µl, 1.14 mmol) and *trans*-[Pt(N_3)$_2$(py)$_2$] (100 mg, 0.229 mmol) were stirred in MeOH (50 ml) at 35 °C for 3 d before the solvent was reduced in volume to 2 ml and placed in the fridge overnight. The solution was filtered and the precipitate washed with cold MeOH to give the title compound as a white solid (17.5 mg, 0.03 mmol, 11 %). NMR spectroscopic experiments were run on freshly made solutions since the complex was unstable in d_6-acetone.

^{1}H NMR (600 MHz, d_6-acetone) δ: 8.67 (d, 4H, $^3J_{HH}$ = 6, H$_o$), 8.03 (dd, 2H, $^3J_{HH}$ = 7, H$_p$), 7.55 (dd, $^3J_{HH}$ = 7, H$_m$), 3.90 (6H, OMe), 3.75 (6H, OMe). **^{14}N NMR** (43 MHz, d_6-acetone) δ: 287.9 (sharp, N_2 gas), 216.1 (br, $W_{1/2}$ 909 Hz $N_{triazole}$), 175.4 (br, $W_{1/2}$ 793 Hz, N_{py}/$N_{triazole}$). **^{195}Pt NMR** (129 MHz, d_6-acetone) δ: -2331 ($W_{1/2}$ 1100 Hz).

ESI-MS (MeOH) *m/z*: (M = *trans*-[Pt($C_6H_6N_3O_4$)$_2$(py)$_2$]): 1465.176 ([2M + Na]$^+$ calcd 1465.230); 1443.137 ([2M + H]$^+$ calcd 1443.248); 744.094 ([M + Na]$^+$ calcd 744.110);

722.045 ([M + H]$^+$ calcd 722.128). **HRMS** (MeOH) *m/z*: 744.1095 ([M + Na]$^+$, $C_{22}H_{22}N_8O_8PtNa$, calcd. 744.1101). **IR** ν cm^{-1} : 1726, 1454, 1209, 1172, 771, 693.

Rearrangement complex 2a''

DMAD (**2**, 14 µl, 0.117 mmol) in MeOH (2 ml) was added dropwise to **1** (50 mg, 0.106 mmol) in MeOH (3 ml). The solution was stirred at 35°C for 7d before being placed on ice. Product **2a''** was isolated as a white precipitate by filtration and rinsed with cold H_2O, MeOH and diethylether (18 mg, 0.03 mmol, 28 %). Complex **2a''** could also be obtained by dissolving **2a'** in $CDCl_3$ and leaving for several days.

^{1}H NMR (500 MHz, $CDCl_3$) δ: 9.01 (d, $^3J_{HPt}$ = 27, $^3J_{HH}$ = 6.0, 4H, H_o), 8.08 (t, $^3J_{HH}$ = 6, J_{HH} = 6, 2H, H_p), 7.66 (dd, $^3J_{HH}$ = 6, $^3J_{HH}$ = 6, 4H, H_m), 3.88 (s, OCH_3, 3H), 2.89 (br, 1H), 1.69 (br, 1H).

^{195}Pt NMR (107 MHz, $CDCl_3$) δ: 764 (PWHH 680 ppm).

^{14}N NMR (29 MHz, $CDCl_3$) δ: 288.6 (N_2 gas), 227.2 (N_β), 168.2 (N_γ/N_{py}). N_α not seen.

^{13}C NMR (125 MHz, $CDCl_3$) δ: 164.3 (ester), 160.8 (ester), 149.4 $C_{pyortho}$), 142.2 (C_{pypara}), 139.1 ($^3J_{CPt}$= 31.0, $C_{triazole}$), 135.6 ($^3J_{CPt}$= 18.0, $C_{triazole}$), 127.1 ($^3J_{CPt}$= 27.0, C_{pymeta}), 52.4 (C_{alkyl}).

IR (solid) ν cm^{-1}: 3465, 2048 ($_{asym}N_3$), 1733, 1675, 1611, 1483, 1437, 1389, 1338, 1265, 1255, 1222, 1211, 1128, 1090, 1079, 836, 774, 691.

ESI-MS identical to **2a'**.

Rearrangement complex 2b''

Complex **2b''** was obtained by dissolving **2b'** in $CDCl_3$ and leaving for several days.

^{1}H NMR (400 MHz, $CDCl_3$) δ: 8.94 (d, $^3J_{HPt}$ = 27.0, $^3J_{HH}$ = 6, 2H, H_o), 8.84 (dd, $^3J_{HPt}$ = 27.0, $^3J_{HH}$ = 6, 2H, $H_{o'}$), 8.10 (t, $^3J_{HH}$ = 6, 1H, H_p), 8.08 (t, $^3J_{HH}$ = 6, 1H, $H_{p'}$), 7.63 (m, $^3J_{HH}$ = 6, 4H, H_m), 3.91 (s, OCH_3, 3H), 3.88 (s, OCH_3, 3H).

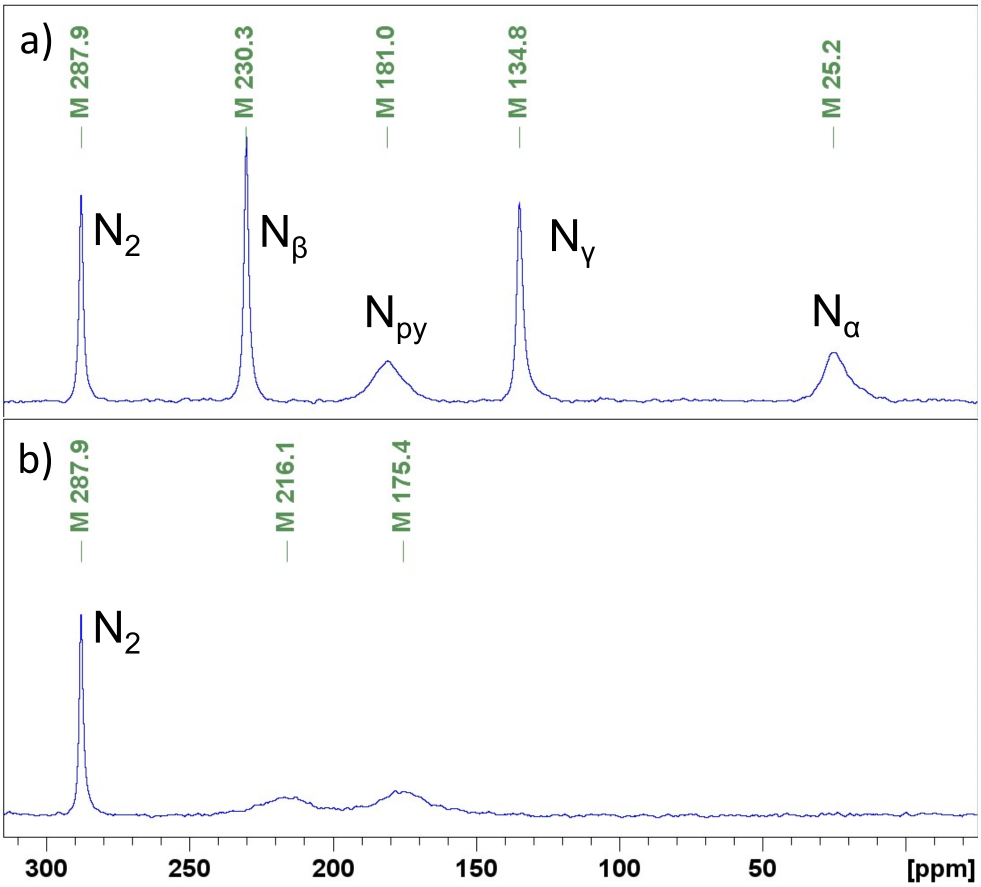

Figure S1. ^{14}N NMR (43 MHz) (d_6-acetone) spectra of a) *trans*-[Pt(N_3)$_2$(py)$_2$] starting material[1] and b) *bis* substituted *trans*-[Pt($C_6H_6N_3O_4$)$_2$(py)$_2$].

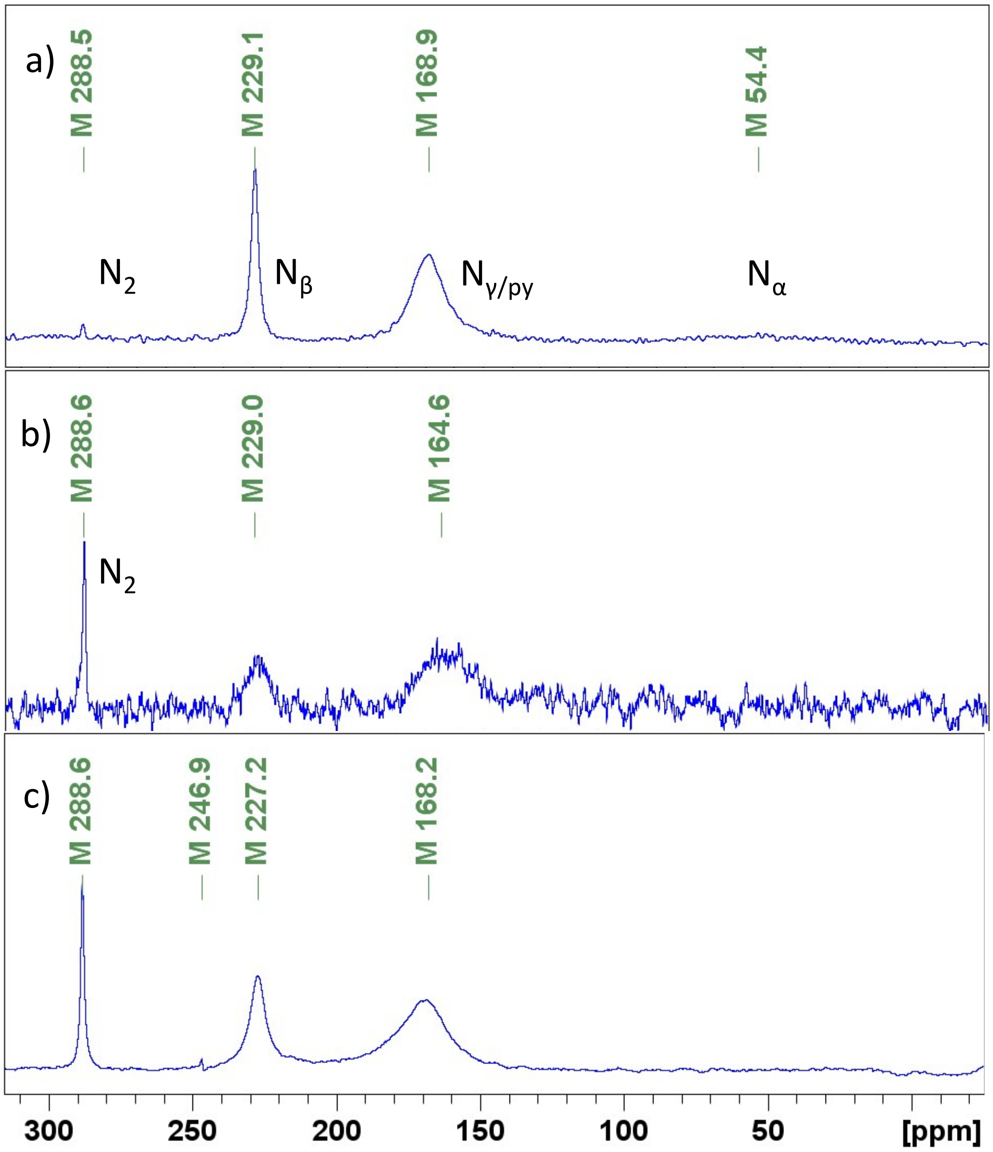

Figure S2. ^{14}N NMR spectra of a) complex **1** (D_2O, 43 MHz) starting material[1] b) complex **2a'** (D_2O, 29 MHz) and c) complex **2a''** ($CDCl_3$, 28 MHz).

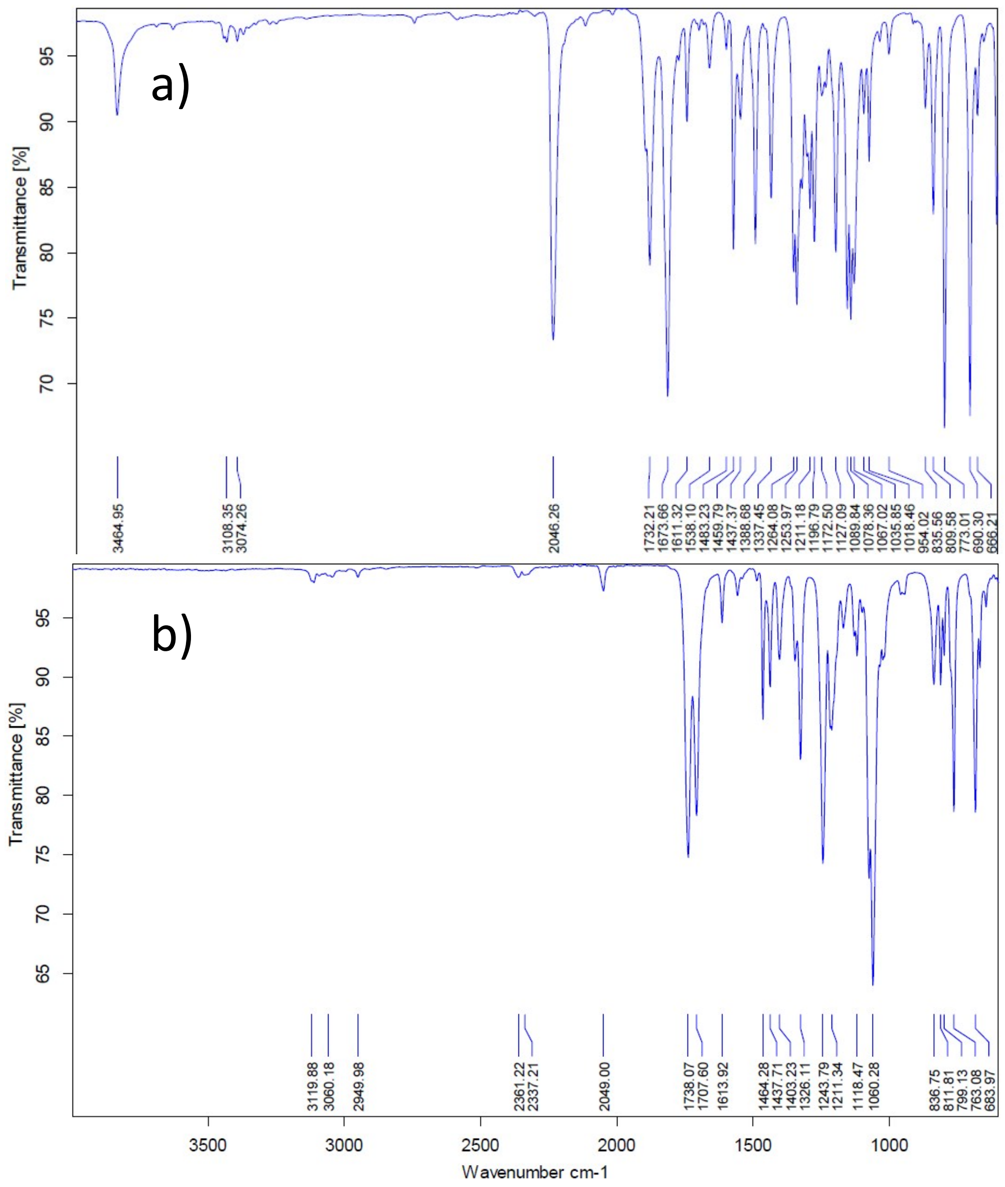

Figure S3. Solid-state IR spectra of a) *mono*-substituted **2a’** and b) *bis*-substituted **2b’**.

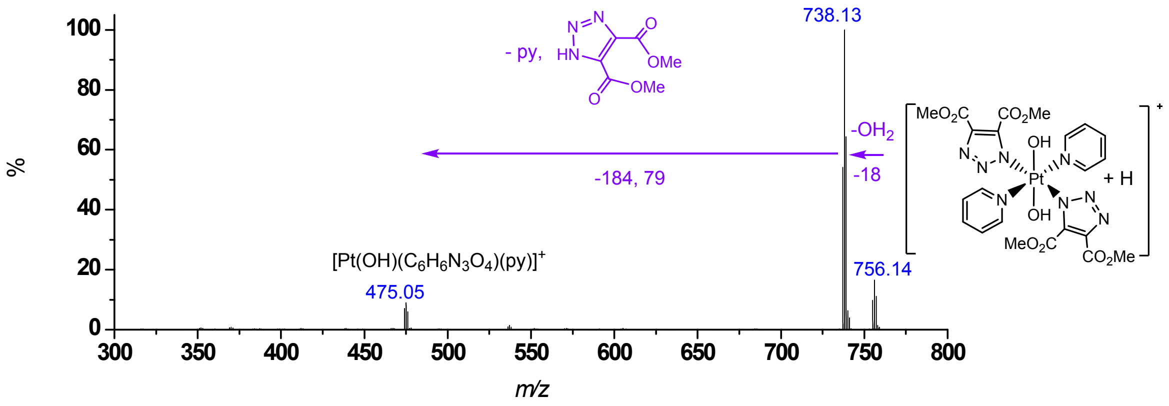

Figure S4. MS/MS of **2b**: *bis*-substituted DMAD complex *trans,trans,trans*-[Pt $(C_6H_6N_3O_4)_2(OH)_2(py)_2$ + H]$^+$, 756.14 *m/z* (model 756.13 *m/z*).

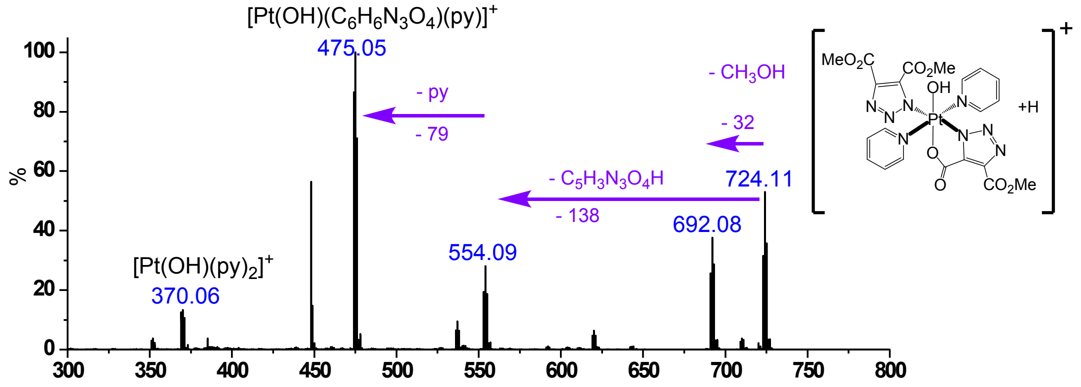

Figure S5. MS/MS of species intermediate between **2b and 2b'**: *bis*-substituted, *mono*-axially tethered DMAD complex *trans,trans,trans*-[Pt$(C_6H_6N_3O_4)_2(C_5H_3N_3O_4)(OH)(py)_2$ + H]$^+$, 724.11 *m/z* (model 724.10 *m/z*).

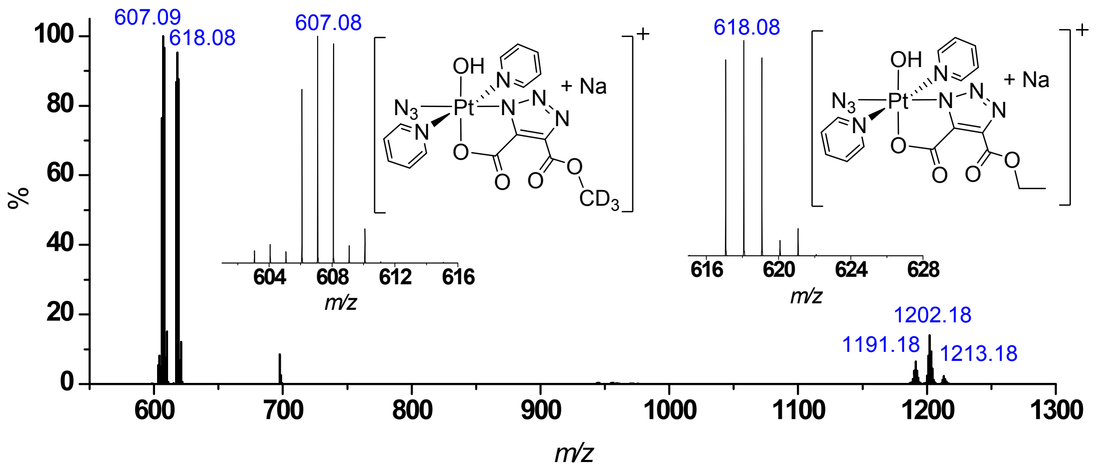

Figure S6. ESI-MS (MeCN) of crystalline DEACD product **3a'** from reaction in d_4-MeOH: a mixture of cyclometallated transesterified species $[Pt(py)_2(N_3)(OH)(N_3C_5O_4D_3) + Na]^+$ at 607.09 *m/z* (model 607.08 *m/z*) and cyclometallated product $[Pt(py)_2(N_3)(OH)(C_6H_5N_3O_4) + Na]^+$ at 618.08 *m/z* (model 618.08 *m/z*).

UV-vis spectra

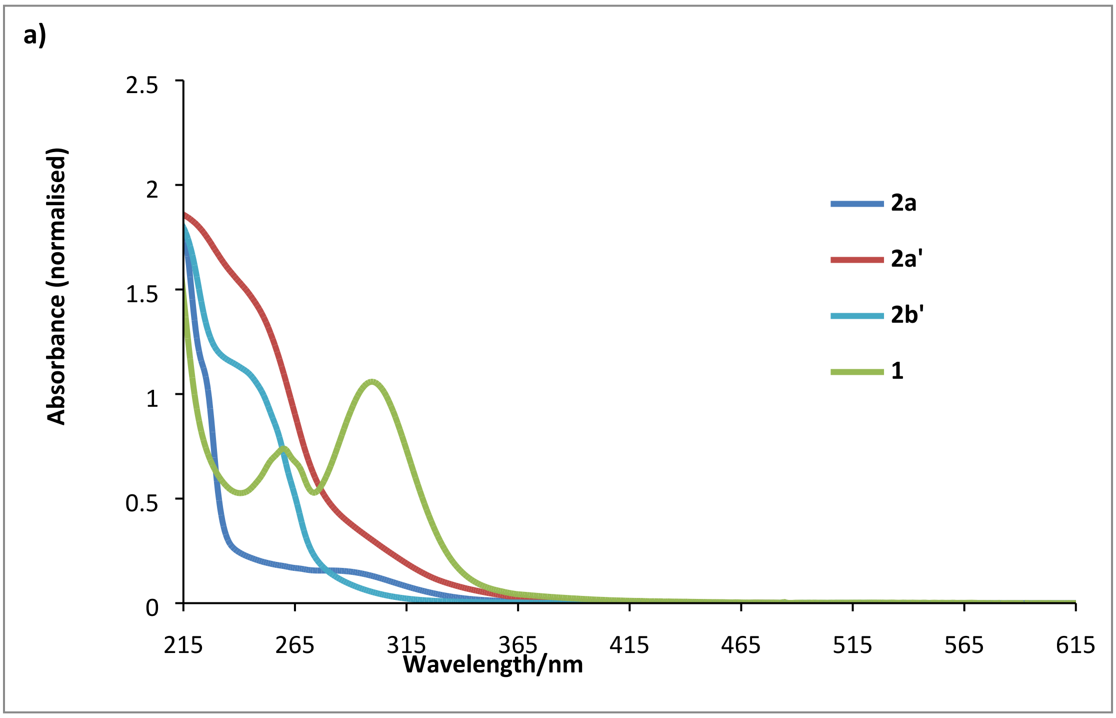

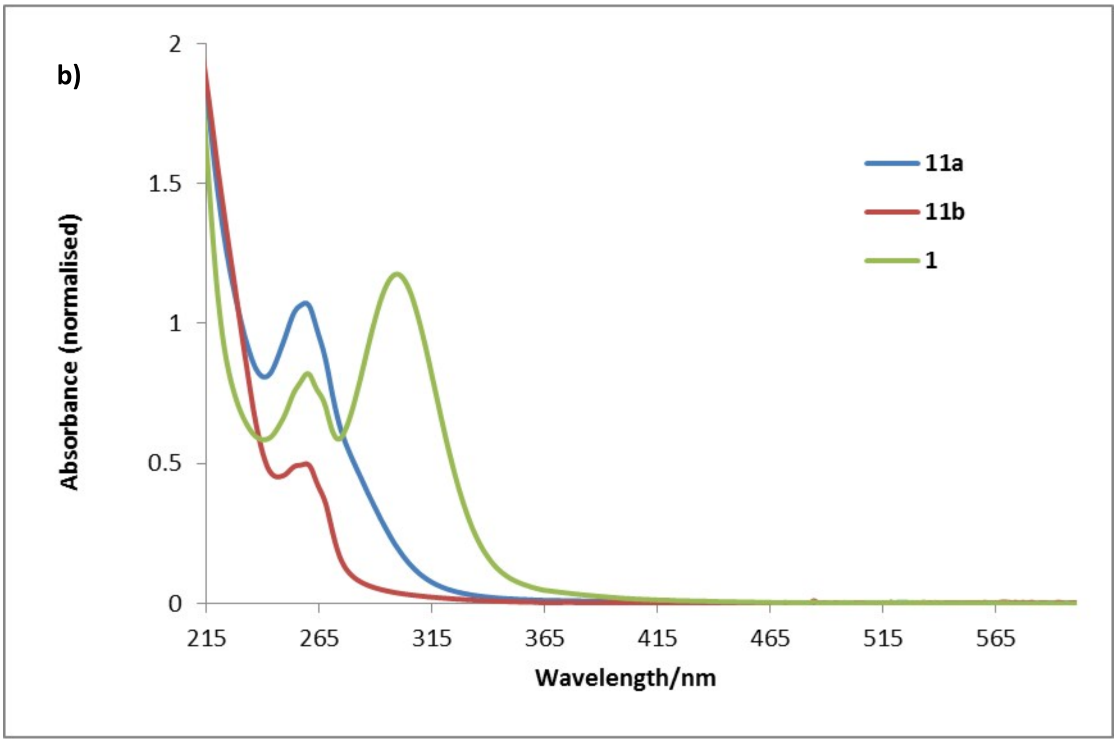

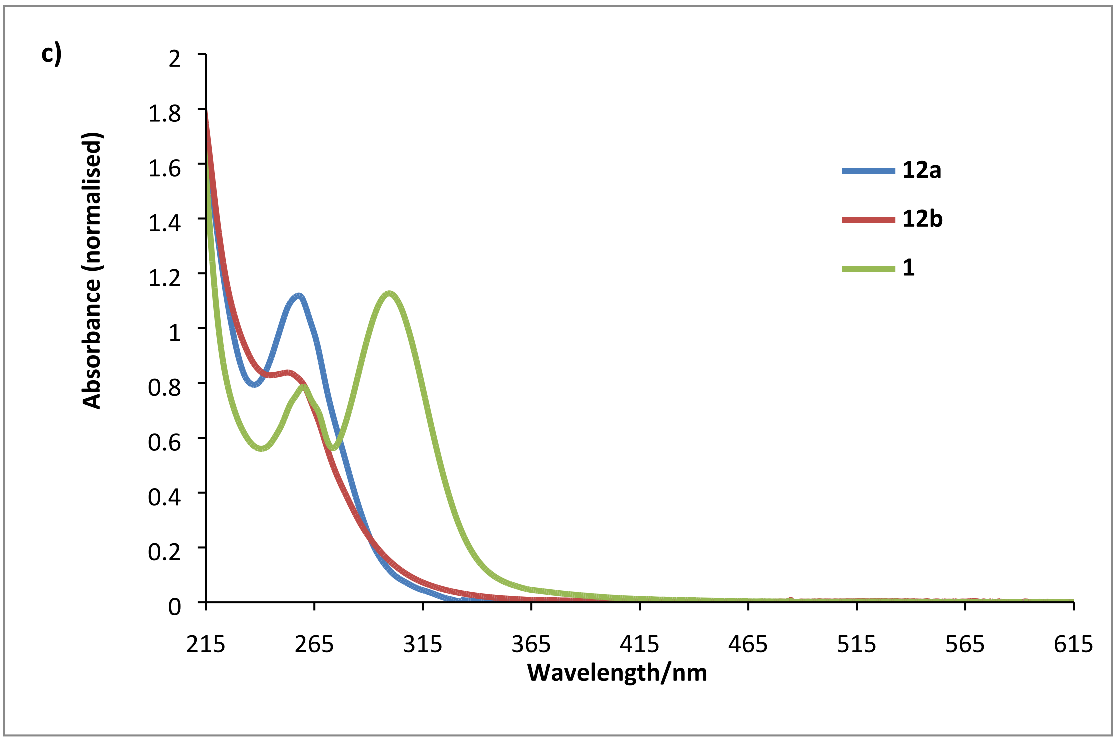

Figure S7. UV-Vis spectra of **1** and its derivatives in MeCN/H_2O, with absorbance normalised at 211 nm for all species: a) DMAD (**2**) derivatives b) BCN (**11**) derivatives c) DBCO (**12**) derivatives

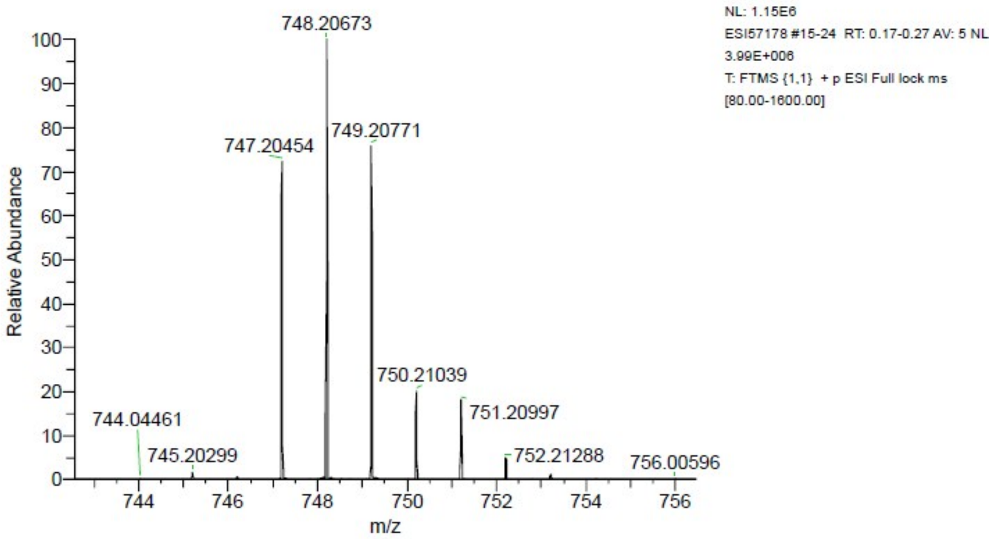

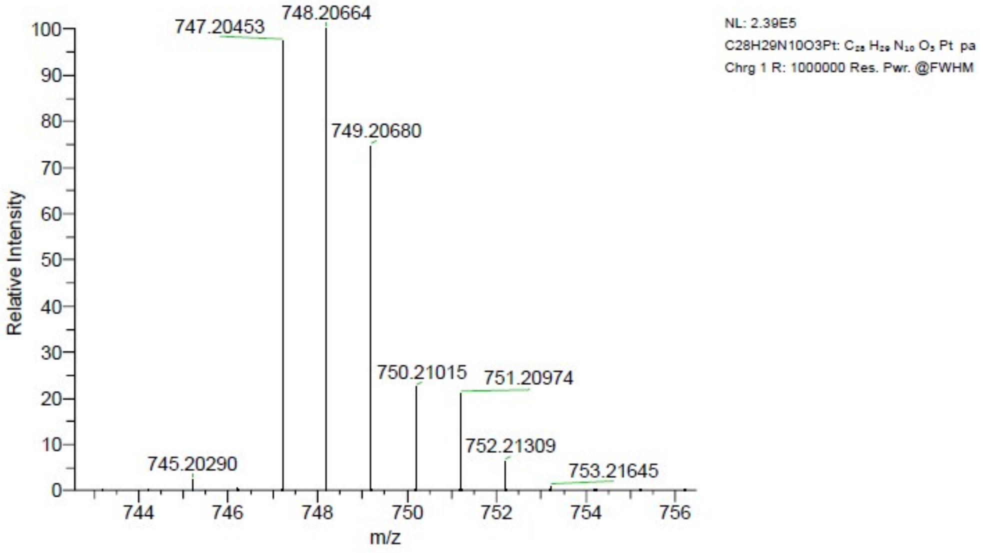

m/z	Formula	RDB	Delta ppm	Theo. Mass
748.20673	$C_{28}H_{29}O_3N_{10}{}^{195}Pt$	19.5	0.12	748.20664

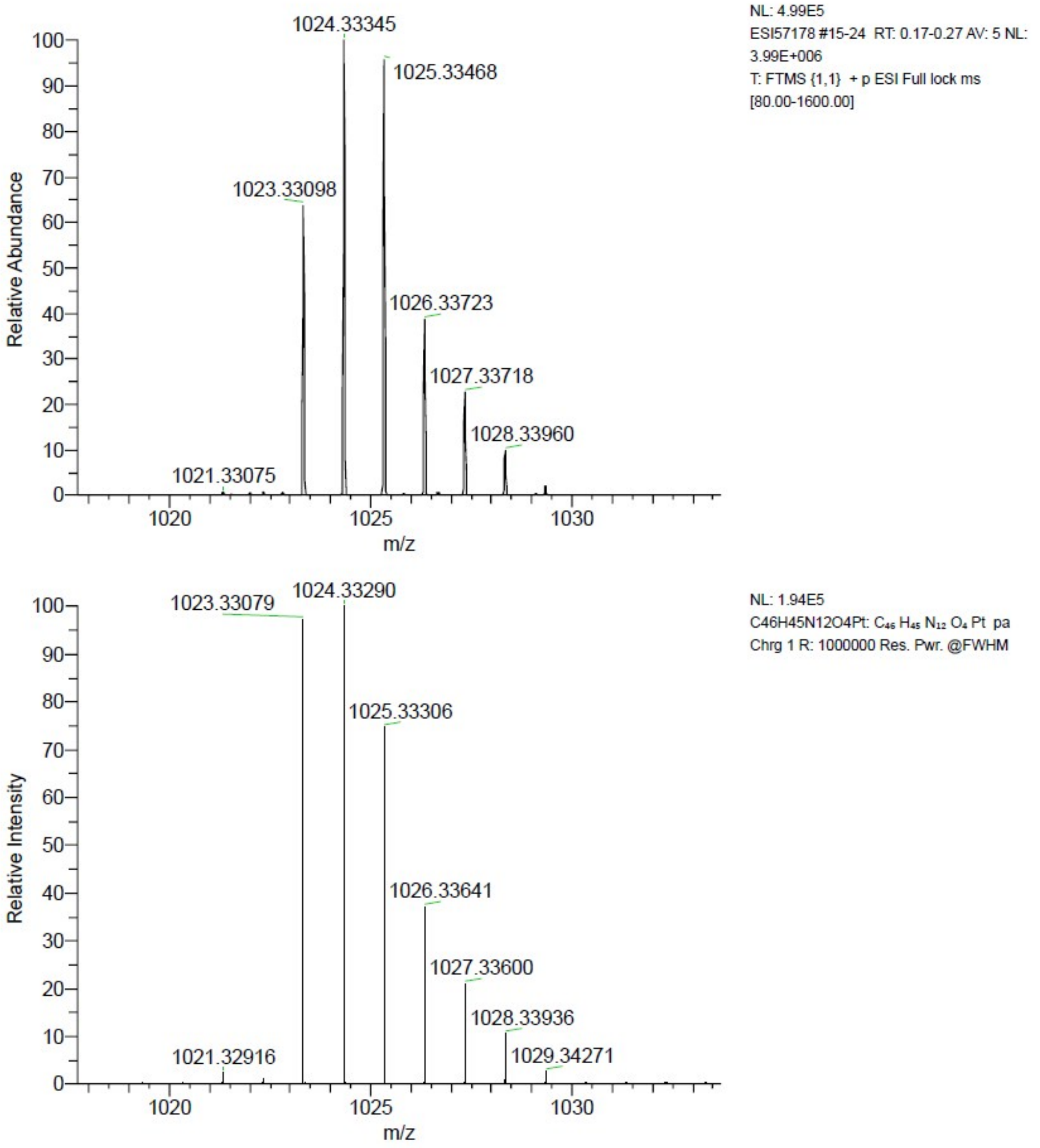

m/z	Formula	RDB	Delta ppm	Theo. Mass
1024.33350	$C_{46}H_{45}O_4N_{12}{}^{195}Pt$	30.5	0.58	1024.33290

Figure S8. HRMS of DBCO products **12a** (748.2067 *m/z*, top) and **12b** (1024.3335 m/z, bottom) with isotope predictions.

References

1 N. J. Farrer, P. Gierth and P. J. Sadler, *Chem. Eur. J.*, 2011, **17**, 12059–66.
